# Enhancing Obstructive Sleep Apnea Diagnosis With Screening Through Disease Phenotypes: Algorithm Development and Validation

**DOI:** 10.2196/25124

**Published:** 2021-06-22

**Authors:** Daniela Ferreira-Santos, Pedro Pereira Rodrigues

**Affiliations:** 1 MEDCIDS-FMUP – Community Medicine, Information and Decision Sciences Faculty of Medicine of the University of Porto Porto Portugal; 2 CINTESIS – Center for Health Technology and Services Research Porto Portugal

**Keywords:** obstructive sleep apnea, screening, risk factors, phenotypes, Bayesian network classifiers

## Abstract

**Background:**

The American Academy of Sleep Medicine guidelines suggest that clinical prediction algorithms can be used in patients with obstructive sleep apnea (OSA) without replacing polysomnography, which is the gold standard.

**Objective:**

This study aims to develop a clinical decision support system for OSA diagnosis according to its standard definition (apnea-hypopnea index plus symptoms), identifying individuals with high pretest probability based on risk and diagnostic factors.

**Methods:**

A total of 47 predictive variables were extracted from a cohort of patients who underwent polysomnography. A total of 14 variables that were univariately significant were then used to compute the distance between patients with OSA, defining a hierarchical clustering structure from which patient phenotypes were derived and described. Affinity from individuals at risk of OSA phenotypes was later computed, and cluster membership was used as an additional predictor in a Bayesian network classifier (model B).

**Results:**

A total of 318 patients at risk were included, of whom 207 (65.1%) individuals were diagnosed with OSA (111, 53.6% with mild; 50, 24.2% with moderate; and 46, 22.2% with severe). On the basis of predictive variables, 3 phenotypes were defined (74/207, 35.7% low; 104/207, 50.2% medium; and 29/207, 14.1% high), with an increasing prevalence of symptoms and comorbidities, the latter describing older and obese patients, and a substantial increase in some comorbidities, suggesting their beneficial use as combined predictors (median apnea-hypopnea indices of 10, 14, and 31, respectively). Cross-validation results demonstrated that the inclusion of OSA phenotypes as an adjusting predictor in a Bayesian classifier improved screening specificity (26%, 95% CI 24-29, to 38%, 95% CI 35-40) while maintaining a high sensitivity (93%, 95% CI 91-95), with model B doubling the diagnostic model effectiveness (diagnostic odds ratio of 8.14).

**Conclusions:**

Defined OSA phenotypes are a sensitive tool that enhances our understanding of the disease and allows the derivation of a predictive algorithm that can clearly outperform symptom-based guideline recommendations as a rule-out approach for screening.

## Introduction

### Background

Obstructive sleep apnea (OSA) is a common sleep-related breathing disorder characterized by clinical symptoms (eg, daytime sleepiness) and at least five events per hour of narrowing (apnea or hypopnea) of the upper airway that impairs normal ventilation during sleep [1]. An apnea consists of a cessation of airflow higher than 90% of the baseline, a hypopnea is a reduction in airflow along with a decreased saturation of 3% from pre-event baseline and/or associated with an arousal, and the apnea-hypopnea index (AHI) is the number of such events per hour of sleep. OSA prevalence has been underestimated, with studies varying significantly, both in the population being studied and in OSA definition. A study using a simpler hypopnea definition (4% desaturation) estimated a prevalence of 14% in men and 5% in women [2]. In 2 other studies, the prevalence was substantially higher but was estimated for specific populations, such as patients being evaluated for bariatric surgery [3] or patients who have had a transient ischemic attack or stroke [4], reaching values of 70% and 72%, respectively. The latest study by Benjafield et al [5] estimated that 936 million adults have OSA; in Portugal, it represents 17%, and approximately 74% have moderate to severe OSA. Overall, this disease is largely unrecognized and undiagnosed, representing a significant burden to the health care system [6], especially for patients who remain untreated or at an increased risk of developing cardiovascular disease, metabolic dysregulation, or diabetes [1,7-11]. The failure to clinically recognize OSA leads to significant morbidity and mortality, making it essential to anticipate its recognition, diagnosis, and treatment [1]. OSA diagnosis, for which a comprehensive sleep evaluation (sleep history and physical examination) plus polysomnography (PSG) is the gold standard [1], can effectively decrease health care utilization and costs, whereas timely treatment can improve quality of life, lower the rates of motor vehicle crashes, and reduce the risk of chronic health consequences [12].

In 2017, a new clinical practice guideline for diagnostic testing for adults with OSA was issued by the American Academy of Sleep Medicine (AASM) [1], updating 2 previous AASM guidelines from 2005 [8] and 2007 [13]. Of the 9 PICO (patient, population or problem, intervention, comparison, and outcome) questions raised in this new guideline, the task force reported insufficient evidence to directly address the first one: “In adult patients with suspected OSA, do clinical prediction algorithms accurately identify patients with a high pretest probability for OSA compared to history and physical exam?,” as no studies comparing the efficacy of clinical prediction algorithms with clinical history and physical examination were identified. Therefore, they compared the efficacy of clinical prediction algorithms with PSG, crafting recommendation 1: “We recommend that clinical tools, questionnaires and prediction algorithms not to be used to diagnose OSA in adults, in the absence of PSG,” affirming that clinical prediction algorithms can, however, be used in patients with suspected OSA, as long as not to establish the need for PSG or to become a substitute for PSG. Rather, these tools can be more helpful, in specialties other than sleep-oriented ones, to identify patients with an increased risk for OSA.

### Objective

In this study, we aim to establish a new clinical prediction algorithm to allow OSA screening (high pretest probability for OSA) based on demographics, physical examination, clinical history, and comorbidities, using standard OSA definition (AHI ≥5 plus symptoms), extending traditional approaches that assess only preestablished symptoms, such as snoring, witnessed apneas, and excessive daytime sleepiness.

## Methods

### Overview

Using retrospective data from a cohort of patients who underwent PSG, after proper referral by a physician, significant predictive variables were selected and used to compute distances among patients with OSA, which supported a clustering algorithm to derive patient phenotypes from resulting clusters, with missing data being analyzed and imputed as needed. To assess the consistency of our phenotypes, each healthy individual was also tested against the clustering structure, and the resulting phenotyping was analyzed. Then, to assess the benefit of this phenotyping strategy, cluster membership was used as an additional predictive variable and included in a Bayesian network classifier, with validity compared with an equivalent classifier without phenotype information, following the 2015 STARD (Standards for Reporting Diagnostic Accuracy Studies) guideline.

### Patients

Data from patients referred to undergo PSG at Vila Nova de Gaia and Espinho Hospital Center Sleep Laboratory were retrospectively collected. Patients who underwent PSG between January and May 2015 were included if they were aged >18 years and were suspected of having OSA. Nonetheless, exclusion criteria included patients already diagnosed (performing positive airway pressure therapies), patients suspected of having another sleep disease, patients with severe lungs or neurological conditions, and pregnant women. In case of multiple examinations of the same patient, the one with the best sleep efficiency was selected. This study was approved by the Ethics Commission of Vila Nova de Gaia and Espinho Hospital Center, in accordance with the Declaration of Helsinki.

### Predictive Variables

An author-performed literature review on PubMed (April 19, 2015) supported the definition of the relevant variables to be collected from medical and/or sleep laboratory records in which the presence or absence of each information was assessed by a physician, resulting in a total of 47 predictive variables, all in accordance with the current and previous OSA guidelines. The search contained “risk factors,” “sleep apnea, obstructive,” and “diagnosis” as MeSH terms, obtaining 1397 articles, of which 47 were used for variable definition (full review description and references used in this phase are not shown for space purposes but can be provided on request). Selected variables included basic demographic data (gender and age), physical examination (BMI, neck and abdominal circumferences, modified Mallampati classification, and craniofacial and upper airway abnormalities), clinical history (daytime sleepiness, snoring, witnessed apneas, gasping and/or choking, sleep fragmentation, nonrepairing sleep, behavior changes, decreased concentration, morning headaches, decreased libido, sleeping body position, sleep efficiency, participation in vehicle crashes, truck driver activity, driving sleepiness, nocturia, alcohol consumption, smoking, coffee intake, use of sedatives before sleep, family history or genetic evidence, and Epworth Sleepiness Scale), and comorbidities and cointerventions (stroke, myocardial infarction, pulmonary infarction, arterial or pulmonary hypertension, congestive heart failure, arrhythmias, respiratory changes, diabetes, dyslipidemia, renal failure, hypothyroidism, gastroesophageal reflux, anxiety and/or depression, insomnia, glaucoma, pacemaker or implantable cardioverter-defibrillator, and bariatric surgery).

### Data Set Description

Clinical data from each patient (47 predictive variables plus the outcome) were extracted from the central clinical data registry (all records were fulfilled by a physician) along with sleep laboratory data and adequately anonymized to ensure patient privacy. Original files included structured demographic data, structured PSG reports, and unstructured textual annotations from the medical records, with many abbreviations and short-form text. The outcome measure was obtained from the AHI, categorized as mild (AHI between 5 and 14), moderate (AHI between 15 and 29), and severe (AHI >30). Given the categorical characteristic of our modeling strategies, all continuous variables were discretized, and the following common cutoffs were extracted from the literature: (1) age (20-44 years, 45-64 years, and 65-90 years), (2) BMI (<25 kg/m^2^ as normal weight, 25-30 kg/m^2^ as overweight, and ≥30 kg/m^2^ as obese), (3) female neck circumference (≤37 cm as normal and >38 cm as increased), (4) male neck circumference (≤41 cm as normal and >42 cm as increased), (5) female abdominal circumference (≤80 cm as normal and >81 cm as increased), (6) male abdominal circumference (≤94 cm as normal and >95 cm as increased), (7) Epworth Sleepiness Scale (0-10 as normal and 11-24 as excessive daytime sleepiness), and (8) AHI (0-4 as normal, 5-14 as mild, 15-29 as moderate, and ≥30 as severe).

### Missing Data Imputation

Although we had all the electronic clinical records from the included patients, after screening all unstructured text reports, some predictive variables were not fully present or described, as physicians normally do not mention the absence of a disease or it could only be noted in paper records (missing data proportions ranged from 0% for gender to 97% for bariatric surgery). In our previous study [14], we studied the impact of missing data imputation, using nearest neighbor (NN) strategies, on the structure learning of Bayesian network classifiers for OSA diagnosis, concluding that it can expand the body of evidence for modeling without compromising validity. In this study, we followed the same strategy: (1) variables with more than 80% missing values were removed from the analysis (ie, behavior changes, decreased libido, decreased concentration, pulmonary infarction, glaucoma, and bariatric surgery); (2) remaining variables were ranked by the proportion of missing values; (3) data imputation started using only complete and outcome-wise statistically significant variables (*P*<.20), imputing incomplete likewise significant variables; and (4) remaining incomplete variables were then imputed stepwise by increasing the proportion of missing values per variable. All imputations were performed using majority voting from the 10 NNs/patients.

### Clinical Prediction Algorithm

Aspiring to a more personalized approach to evaluate patients with OSA and targeting to recognize high pretest probability for OSA, cluster analysis (a statistical approach for studying the relationship present among groups of patients or variables [7]) was applied to distinguish whether there are different subgroups of patients with different clinical presentations, that is phenotypes. Clustering has been widely used in health research, particularly in the analysis of gene expression [15], asthma [16], chronic obstructive pulmonary disease [17], fibromyalgia [18], Parkinson disease [19], and sleep apnea [20-22]. The aim is to identify clusters of patients who are similar among themselves, although significantly different from patients of other clusters [7]. As expected, different clusters created from predictive variables express different disease risks, hence defining risk-aligned phenotypes.

### Connectivity-Based Clustering

In this study, we applied a hierarchical clustering algorithm to obtain a hierarchy of possible solutions, ranging from one single group with all patients to having every single patient separated from each other. This process, where a cluster hierarchy is created, is based on the distance between data observations (ie, patients), giving as output a dendrogram (a tree diagram that presents different clustering definitions for all possible numbers of clusters, from which the user might choose the desired number of clusters after inspecting the intracluster and intercluster distances of each possible cut point). Therefore, the definition of the distance function is a crucial step in the application of this technique, especially in categorical data, as an incorrect distance can easily lead to biased results with potentially serious consequences to the conclusions drawn.

In this study, we computed the distance measure between 2 patients, a and b, based only on significant variables (univariate significant association with the outcome, for a 20% significance level in both the original and imputed data sets, using chi-square and Fisher exact tests), and each variable was weighted according to the corresponding crude odds ratio for the severe level, as follows:

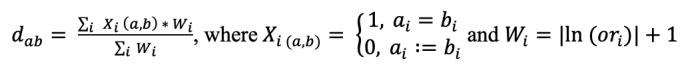



This distance encoded the similarity between patients weighted by the contribution of each variable toward the outcome, regularized for significant variables only, and was subsequently used in hierarchical clustering with Ward linkage, leading to a complete dendrogram. Afterward, the obtained OSA clusters were defined by inspecting the outcome proportion by cluster and the corresponding 95% CIs.

### Phenotypes Consistency

To assess whether predetermined phenotypes would also help in segmenting healthy patients, each healthy patient was assigned to the closest phenotype using the aforementioned distance measure and the same significant variables, determining the distance between each healthy patient and obtained OSA cluster. The resulting clustering definition was then described and analyzed, as was done for the cohort of patients with OSA.

### Phenotypes Predictive Value

To assess whether the phenotypes could encode any predictive value, Bayesian network classifiers were built with and without cluster information as a predictive variable. First, a naïve Bayesian network classifier was induced using the selected variables. Then, assigned cluster was also included in the model as a parent node of all independent variables. Validity was then assessed and compared using leave-one-out and 10 times twofold cross-validation strategies, comparing validity measures, such as sensitivity, specificity, accuracy, predictive values, area under the receiver operating characteristic (ROC) curve, likelihood ratios, posttest odds and posttest probabilities, and diagnostic odds ratio.

### Statistical Software

R 3.2.2 (R Development Core Team) software [23] was used on every statistical step of this work: discretization of continuous variables (package car [24]), descriptive and comparative analyses (packages gmodels [25] and epitools [26]), missing data analysis (package summarytools [27]), missing data imputation (package DMwR [28]), hierarchical clustering (package stats [23]), Bayesian network inference (packages bnlearn [29] and gRain [30]), and ROC curve analysis (package pROC [31]). Bayesian networks were visually inspected using SamIam software (developed by the University of California, Los Angeles) [32].

## Results

### Baseline Characteristics

Of the 318 patients included, 207 (65.1%) had OSA. Of these 207 patients, 111 (53.6%) were classified as mild, 50 (24.2%) as moderate, and 46 (22.2%) as severe. Baseline characteristics of patients with OSA and the proportion of missing values for each predictive variable are described below in Table 1 (original data) and in Multimedia Appendix 1 (for the curated data, after missing data imputation).

Patients with OSA had a mean age of 61 (SD 11) years, being slightly older in the moderate subgroup (24/50, 48%; aged >65 years), whereas the proportion of males was higher in the moderate (40/50, 80%) and severe (35/46, 76%) subgroups. Beyond these 2 variables, only sleep efficiency was found to be complete (no missing data), and no differences were found across OSA levels (*P*=.65). For the remaining variables, distributions were computed before and after data imputation.

The presence of witnessed apneas (109/169, 64.5%), nonrepairing sleep (93/183, 50.8%), nocturia (99/136, 72.8%), stroke (23/44, 52%), arterial hypertension (136/159, 85.5%), diabetes (62/99, 63%), and dyslipidemia (125/148, 84.5%) were more prevalent in patients with OSA than in healthy patients, whereas the opposite was observed in family history (14/77, 18%), pulmonary hypertension (15/117, 12.8%), congestive heart failure (26/138, 18.8%), arrhythmias (17/99, 17%), pacemaker or implantable cardioverter-defibrillator (10/91, 11%), and respiratory changes (81/185, 43.8%). After data imputation, the same variables remained different across OSA levels, except for family history. Only variables significantly associated with the outcome (*P*<.20) on both the original and curated data sets were further considered for the clustering process.

**Table 1:** Descriptive analysis of patients with obstructive sleep apnea (absolute and relative frequencies are presented, and *P* values are the results of chi-square tests unless otherwise specified).

Characteristic			Mild (n=111), n (%)		Moderate (n=50), n (%)		Severe (n=46), n (%)		Total (N=207), n (%)		*P* value		Missing, n (%)
Gender (male)			72 (64.9)		40 (80.0)		35 (76.1)		147 (71.0)		*.10* ^a^		207 (0.0)
**Age (years)**											*.18* ^b^		207 (0.0)
	20-44	7 (6.3)		5 (10.0)		6 (13.0)		18 (8.7)					
	45-64	67 (60.4)		21 (42.0)		24 (52.2)		112 (54.1)					
	65-90	37 (33.3)		24 (48.0)		16 (34.8)		77 (37.2)					
**BMI (kg/m^2^)**											*.05* ^b^		169 (18.4)
	Normal weight	14 (15.6)		1 (2.6)		1 (2.5)		16 (9.5)					
	Overweight	34 (37.8)		21 (53.8)		16 (40.0)		71 (42.0)					
	Obesity	42 (46.7)		17 (43.6)		23 (57.5)		82 (48.5)					
Increased neck circumference			50 (64.1)		23 (67.6)		19 (70.4)		92 (66.2)		.82		139 (32.9)
Increased abdominal circumference			48 (87.3)		23 (95.8)		21 (100.0)		92 (92.0)		.22^b^		100 (51.7)
**Modified Mallampati**											.44		142 (31.4)
	Class I	19 (23.2)		3 (10.0)		5 (16.7)		27 (19.0)					
	Class II	29 (35.4)		15 (50.0)		9 (30.0)		53 (37.3)					
	Class III	29 (35.4)		9 (30.0)		12 (40.0)		50 (35.2)					
	Class IV	5 (6.1)		3 (10.0)		4 (13.3)		12 (8.5)					
Craniofacial and upper airway abnormalities			42 (84.0)		15 (83.3)		6 (66.7)		63 (81.8)		.49^b^		77 (62.8)
Daytime sleepiness			61 (55.5)		27 (60.0)		21 (50.0)		109 (55.3)		.64		197 (4.8)
Snoring			103 (92.8)		43 (93.5)		41 (93.2)		187 (93.0)		>.99^b^		201 (2.9)
Witnessed apneas			55 (58.5)		30 (76.9)		24 (66.7)		109 (64.5)		*.12*		169 (18.4)
Gasping and/or choking			39 (45.3)		12 (36.4)		16 (45.7)		67 (43.5)		.65		154 (25.6)
Sleep fragmentation			55 (73.3)		22 (68.8)		19 (73.1)		96 (72.2)		.88		133 (35.7)
Nonrepairing sleep			47 (47.5)		27 (62.8)		19 (46.3)		93 (50.8)		*.20*		183 (11.6)
Morning headaches			34 (46.6)		14 (48.3)		17 (53.1)		65 (48.5)		.83		134 (35.3)
**Body position**											.36^b^		201 (2.9)
	Decubitus	5 (4.5)		0 (0.0)		1 (2.3)		6 (3.0)					
	Left lateral	20 (18.2)		8 (17.0)		8 (18.2)		36 (17.9)					
	Right lateral	56 (50.9)		22 (46.8)		16 (36.4)		94 (46.8)					
	Supine	29 (26.4)		17 (36.2)		19 (43.2)		65 (32.3)					
Bad sleep efficiency			68 (61.3)		28 (56.0)		30 (65.2)		126 (60.9)		.65		207 (0.0)
Vehicle crashes			7 (20.6)		0 (0.0)		3 (20.0)		10 (16.4)		.28^b^		61 (70.5)
Truck driver			5 (4.7)		5 (10.4)		4 (9.5)		14 (7.1)		.32^b^		197 (4.8)
Driving sleepiness			5 (8.9)		4 (17.4)		4 (18.2)		13 (12.9)		.38^b^		101 (51.2)
Nocturia			47 (64.4)		20 (69.0)		32 (94.1)		99 (72.8)		*.005*		136 (34.3)
Alcohol consumption			61 (66.3)		29 (70.7)		29 (74.4)		119 (69.2)		.64		172 (16.9)
**Smoking**											.74		204 (1.4)
	Yes	11 (10.0)		7 (14.6)		5 (10.9)		23 (10.9)					
	Ex-smoker	38 (34.5)		20 (41.7)		17 (37.0)		75 (36.8)					
Coffee intake			77 (87.5)		30 (83.3)		25 (86.2)		132 (86.3)		.85^b^		153 (26.1)
Use of sedatives			23 (22.8)		13 (29.5)		7 (16.3)		43 (22.9)		.34		188 (9.2)
Family history			8 (18.2)		1 (5.9)		5 (31.2)		14 (18.2)		*.18* ^b^		77 (62.8)
Epworth Sleepiness Scale			33 (37.5)		17 (44.7)		10 (29.4)		60 (37.5)		.41		160 (22.7)
Stroke			9 (37.5)		8 (80.0)		6 (60.0)		23 (52.3)		*.08* ^b^		44 (78.7)
Myocardial infarction			9 (12.7)		3 (9.7)		6 (20.0)		18 (13.6)		.52^b^		132 (36.2)
Arterial hypertension			67 (79.8)		32 (91.4)		37 (92.5)		136 (85.5)		*.09*		159 (23.2)
Pulmonary hypertension			5 (8.3)		3 (10.0)		7 (25.9)		15 (12.8)		*.08* ^b^		117 (43.5)
Congestive heart failure			7 (9.9)		6 (17.6)		13 (39.4)		26 (18.8)		*.002*		138 (33.3)
Arrhythmias			5 (10.0)		4 (16.7)		8 (32.0)		17 (17.2)		*.06* ^b^		99 (52.2)
Pacemaker and/or cardioverter			3 (6.1)		2 (9.5)		5 (23.8)		10 (11.0)		*.09* ^b^		91 (56.0)
Respiratory changes			43 (43.0)		15 (32.6)		23 (59.0)		81 (43.8)		*.05*		185 (10.6)
Diabetes			28 (51.9)		12 (60.0)		22 (88.0)		62 (62.6)		*.008*		99 (52.2)
Dyslipidemia			63 (78.8)		28 (90.3)		34 (91.9)		125 (84.5)		*.11*		148 (28.5)
Renal failure			10 (27.0)		6 (50.0)		7 (36.8)		23 (33.8)		.33		68 (67.1)
Hypothyroidism			12 (25.5)		6 (37.5)		6 (35.3)		24 (30.0)		.58		80 (61.4)
Gastroesophageal reflux			22 (48.9)		10 (71.4)		7 (53.8)		39 (54.2)		.34		72 (65.2)
Anxiety and/or depression			41 (78.8)		23 (92.0)		17 (77.3)		81 (81.8)		.31^b^		99 (52.2)
Insomnia			25 (71.4)		10 (76.9)		10 (90.9)		45 (76.3)		.48^b^		59 (71.5)

^a^*P*<.20 are italicized.

^b^Fisher exact test.

### OSA Clusters

Using the 14 variables significantly associated with the outcome, a hierarchical clustering structure was derived, where, given the resulting clustering structure, a 10-cluster cutoff point was chosen (following the hierarchical structure of the clustering in the dendrogram). The resulting clusters had median AHI values of 8, 10 (4 clusters), 12, 13, 14, 31, and 34. As 10 clusters are difficult to interpret in a medical context, we chose to aggregate the 10 created clusters into 3 clusters according to their median values: (1) clusters with median 8 and 10, (2) clusters with median 12, 13, and 14, and (3) clusters with median 31 and 34.

The OSA cluster characteristics of the 14 predictive variables are described below and listed in Table 2. The witnessed apneas variable was also statistically significant in both the original and the curated data but was not considered for the cluster hierarchy, as it depends on third-party reporting, which might create a strong bias in the analysis.

**Table 2:** Clinical characteristics of the obstructive sleep apnea cohort by the defined clusters (*P* values are the results of chi-square tests unless otherwise specified).

Characteristics			Cluster 1 (n=74)					Cluster 2 (n=104)					Cluster 3 (n=29)					*P* value	
			Patient, n (%)		95% CI		Patient, n (%)			95% CI		Patient, n (%)			95% CI				
Gender (male)			51 (68.9)		57-79		72 (69.2)			60-78		24 (82.8)			64-93		.32		
**Age (years)**																	<.001^a^		
	20-44	6 (8.1)		3-17		12 (11.5)			6-20		0 (0.0)			0-15					
	45-64	46 (62.2)		50-73		59 (56.7)			47-66		7 (24.1)			11-44					
	65-90	22 (29.7)		20-42		33 (31.7)			23-42		22 (75.9)			56-89					
**BMI (kg/m^2^)**																	<.001^a^		
	Normal weight	13 (17.6)		10-29		3 (2.9)			1-9		0 (0.0)			0-15					
	Overweight	15 (20.3)		12-32		50 (48.1)			38-58		9 (31.0)			16-51					
	Obesity	46 (62.2)		50-73		51 (49.0)			39-59		20 (69.0)			49-84					
Nonrepairing sleep			34 (45.9)		34-58		57 (54.8)			45-65		10 (34.5)			19-54		.13		
Nocturia			14 (18.9)		11-30		104 (100.0)			96-100		29 (100.0)			85-100		<.001		
Stroke			34 (45.9)		34-58		88 (84.6)			76-91		27 (93.1)			76-99		<.001		
Arterial hypertension			54 (73.0)		61-82		96 (92.3)			85-96		29 (100.0)			85-100		<.001^a^		
Pulmonary hypertension			9 (12.2)		6-22		2 (1.9)			0-8		6 (20.7)			9-40		.002^a^		
Congestive heart failure			4 (5.4)		2-14		1 (1.0)			0-6		23 (79.3)			60-91		<.001^a^		
Arrhythmias			4 (5.4)		2-14		1 (1.0)			0-6		12 (41.4)			24-61		<.001^a^		
Pacemaker and/or cardioverter			0 (0.0)		0-6		4 (3.8)			1-10		6 (20.7)			9-40		<.001^a^		
Respiratory changes			35 (47.3)		36-59		28 (26.9)			19-37		18 (62.1)			42-79		.001		
Diabetes			37 (50.0)		39-61		69 (66.3)			56-75		29 (100.0)			85-100		<.001		
Dyslipidemia			57 (77.0)		66-86		94 (90.4)			83-95		29 (100.0)			85-100		.003^a^		
**Apnea-hypopnea index**																	<.001		
	Mild	51 (68.9)		57-79		54 (51.9)			42-62		6 (20.7)			9-40					
	Moderate	18 (24.3)		15-36		24 (23.1)			16-33		8 (27.6)			13-48					
	Severe	5 (6.8)		3-16		26 (25.0)			17-35		15 (51.7)			33-70					

^a^Fisher exact test.

As shown in Table 2, 68.9% (51/74) of the patients in cluster 1 (74/207, 35.7%) were male, 62.2% (46/74) were aged between 45 and 64 years, and 62.2% (46/74) were obese. Nonrepairing sleep was reported in almost half of the patients, and only 18.9% (14/74) reported nocturia. The occurrence of stroke (34/74, 45.9%) did not reach half of the patients, whereas arterial hypertension (54/74, 73.0%) and dyslipidemia (57/74, 77.0%) surpassed it. Pulmonary hypertension, congestive heart failure, arrhythmias, and pacemaker or implantable cardioverter-defibrillator had percentages lower than 15%. The median AHI was 10 (range 7-17), the lowest AHI value, with 69.8% (44/169) reporting witnessed apneas.

Cluster 2 (104/207, 50.2%) had 69.2% (72/104) of males (the same as cluster 1), and only 2.9% (3/104) had normal weight. In contrast to cluster 1, 100.0% (104/104) of patients reported nocturia, 84.6% (88/104) reported stroke, 92.3% (96/104) reported arterial hypertension, and 90.4% (94/104) reported dyslipidemia. Similar to cluster 1, pulmonary hypertension, congestive heart failure, arrhythmias, and pacemaker or implantable cardioverter-defibrillator had percentages lower than 15%. Respiratory changes were reported in 26.9% (28/104) of the patients, and diabetes was reported in 66.3% (69/104) of the patients, compared with cluster 1. Regarding the clinical outcome, this cluster had a median AHI of 14 (range 8-30). Concerning witnessed apneas, cluster 2 had a percentage of 57.8% (52/169), the lowest value of all 3 clusters.

Cluster 3 (29/207, 14.0%) included the highest percentage of men (24/29, 82.8%). None of the patients were aged between 20 and 44 years or had normal weight. This cluster had the lowest proportion of patients aged between 45 and 64 years; nevertheless, it reached the highest proportion of all clusters in patients aged between 65 and 90 years. Although it had one of the lowest proportions of overweight patients, this cluster had the highest percentage (20/29, 69.0%) of patients with obesity. In contrast to cluster 1, but in concordance with cluster 2, nocturia was described in all patients in cluster 3. In addition, arterial hypertension, diabetes, and dyslipidemia were observed in all the patients. The median AHI was 31 (range 21-60); therefore, it was the highest in all 3 clusters. Witnessed apneas were found with the highest proportion of all clusters (13/169, 81.2%).

Age strata and BMI were found to be different among clusters (*P*<.001). Comorbidities, such as stroke, arterial hypertension, diabetes (*P*<.001), and dyslipidemia (*P=*.003), were increasingly more prevalent from cluster 1 to clusters 2 and 3. Only male sex (*P*=.32) and nonrepairing sleep (*P*=.13) were not found to be significantly different.

On the basis of the description of clusters mentioned earlier, the OSA phenotypes can be defined. We classified patients into low (cluster 1), medium (cluster 2), and high (cluster 3) severity phenotypes, as their median AHI corresponded to mild, moderate, and severe levels respectively, defined in PSG for OSA diagnosis. The low severity phenotype includes age >45 years, a fair distribution in normal and overweight patients, accentuating obesity, and low prevalence of symptoms and comorbidities, except for dyslipidemia and arterial hypertension. The medium severity phenotype has almost the same distribution in age as the low severity phenotype, but less normal-weight patients and more overweight patients. Symptoms and comorbidities were higher, with stroke, arterial hypertension, dyslipidemia, and nocturia appearing in more than 85% of the patients with this phenotype. The high severity phenotype presents older and obese patients, with additional comorbidities (congestive heart failure and diabetes) beyond those present in the medium severity phenotype. The foremost difference between our phenotypes and AHI alone is that we considered the risk and diagnostic factors associated with the patient and not only a single value or a counting of events.

### Affinity Between Healthy Patients and OSA Phenotypes

Given that our data set included patients who are healthy and with OSA (a total of 318 individuals), we focused our attention on exploring whether the determined OSA phenotypes could also help to segment healthy patients. To do so, we computed the aforementioned distance measure between 2 individuals using the same 14 significant variables. Table 3 describes the baseline characteristics of healthy patients for each OSA phenotype.

As expected, a high severity phenotype was less common in healthy patients (7/111, 6.3%), including older (*P*<.001), females (*P*=.49), and obese individuals (*P*=.50), with a lower proportion of individuals reporting nonrepairing sleep (*P*=.36). This phenotype also presented the highest proportion of reported nocturia, stroke, arterial hypertension, congestive heart failure, and diabetes (*P*<.001); pulmonary hypertension and arrhythmias (*P*=.01); and respiratory changes (*P*=.11). The medium severity phenotype had the highest proportion of overweight males aged between 45 and 64 years. Although comorbidities such as pulmonary hypertension, congestive heart failure, arrhythmias, and pacemaker or implantable cardioverter-defibrillator do not reach proportions higher than 1%, others such as stroke, arterial hypertension, diabetes, and dyslipidemia present proportions higher than 70%. The low severity phenotype is similar to the medium severity phenotype in terms of the proportion of overweight males, but individuals are younger. Nocturia, pulmonary hypertension, congestive heart failure, arrhythmias, pacemaker or implantable cardioverter-defibrillator, and diabetes have not been reported in this phenotype. Dyslipidemia was the most common comorbidity (16/25, 64%), followed by arterial hypertension (14/25, 56%) and respiratory changes (7/25, 28%).

**Table 3:** Clinical characteristics of the healthy cohort by the predefined obstructive sleep apnea phenotypes (*P* values are the result of chi-square test, unless otherwise specified).

Characteristics			Low OSA^a^ (n=25)					Medium OSA (n=79)					High OSA (n=7)					*P* value^b^
			Patient, n (%)		95% CI		Patient, n (%)			95% CI		Patient, n (%)			95% CI			
Gender (male)			10 (40)		22-61		39 (49)			38-61		2 (29)			5-70		.49	
**Age (years)**																	<.001	
	20-44	16 (64)		43-81		15 (19)			11-30		0 (0)			0-44				
	45-64	7 (28)		13-50		47 (59)			48-70		2 (29)			5-70				
	65-90	2 (8)		1-28		17 (22)			13-32		5 (71)			30-95				
**BMI**																	.50	
	Normal weight	4 (16)		5-37		6 (8)			3-16		1 (14)			1-58				
	Overweight	11 (44)		25-65		40 (51)			39-62		2 (29)			5-70				
	Obesity	10 (40)		22-61		33 (42)			31-53		4 (57)			20-88				
Nonrepairing sleep			18 (72)		50-87		51 (65)			53-75		3 (43)			12-80		.36	
Nocturia			0 (0)		0-17		54 (68)			57-78		6 (86)			42-99		<.001	
Stroke			1 (4)		0-22		66 (84)			73-91		6 (86)			42-99		<.001	
Arterial hypertension			14 (56)		35-75		78 (99)			92-100		7 (100)			56-100		<.001	
Pulmonary hypertension			0 (0)		0-17		1 (1)			0-8		2 (29)			5-70		.01	
Congestive heart failure			0 (0)		0-17		0 (0)			0-6		7 (100)			56-100		<.001	
Arrhythmias			0 (0)		0-17		1 (1)			0-8		2 (29)			5-70		.01	
Pacemaker and/or cardioverter			0 (0)		0-17		1 (1)			0-8		0 (0)			0-44		>.99	
Respiratory changes			7 (28)		13-50		34 (43)			32-55		5 (71)			30-95		.11	
Diabetes			0 (0)		0-17		55 (70)			58-79		7 (100)			56-100		<.001	
Dyslipidemia			16 (64)		43-81		75 (95)			87-98		6 (86)			42-99		.001	

^a^OSA: obstructive sleep apnea.

^b^Fisher exact test.

### Beyond OSA Phenotypes

OSA is a systemic disorder that remains underdiagnosed. Physicians, particularly nonspecialists in sleep disorders, urgently need a simple yet complete tool that allows them to identify a high pretest probability for OSA. This ability, which could enhance current screening, could lead to personalized treatment by additionally improving the understanding of OSA mechanisms and the risk for adverse events.

Our clinical prediction algorithm, that is, previously described OSA phenotypes, is a new way to screen patients, extending traditional approaches. To implement this new strategy, we need a simple, understandable, and updatable tool that can be used daily and that takes into account the knowledge of experts, the literature evidence, and the clinical data.

Belief or Bayesian networks [33] are probabilistic graphical models used to represent knowledge about an uncertain domain; each node represents a random variable, whereas directed edges between the nodes represent probabilistic dependencies among the corresponding variables. Bayesian networks are both mathematically rigorous and intuitively understandable, as they reflect a simple conditional independence statement, that is, each variable is independent of its nondescendants in the graph, given the state of its parents. The Bayesian network thus consists of both a qualitative model (which shows the relationship among variables) and a quantitative model (the joint probability distribution is expressed as conditional probabilities).

**Figure 1:**
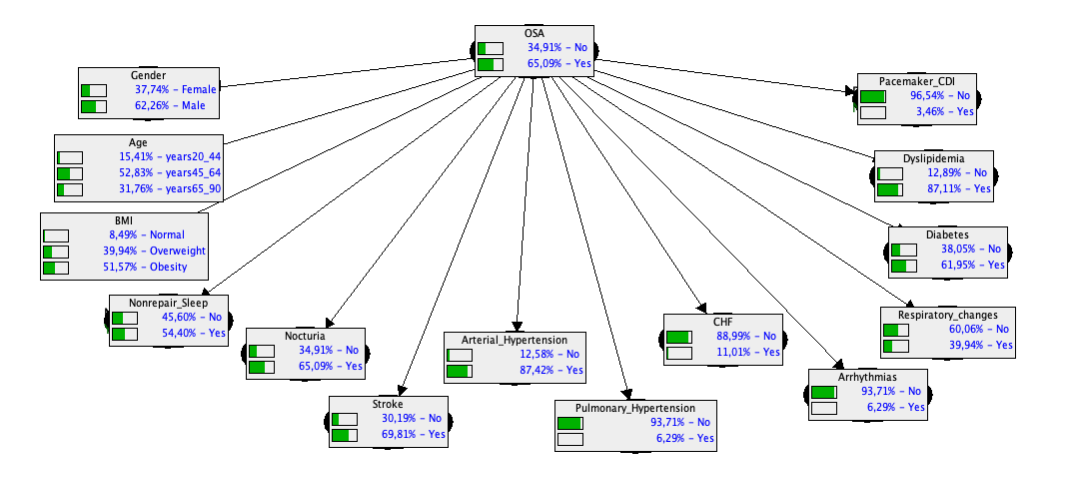
Naïve Bayesian network representation of the relationships between the outcome (obstructive sleep apnea) and each of the 14 significant predictive variables. The bars within each variable represent the prior marginal probabilities for the category of each variable. CDI: implantable cardioverter-defibrillator; CHF: congestive heart failure; OSA: obstructive sleep apnea.

**Figure 2:**
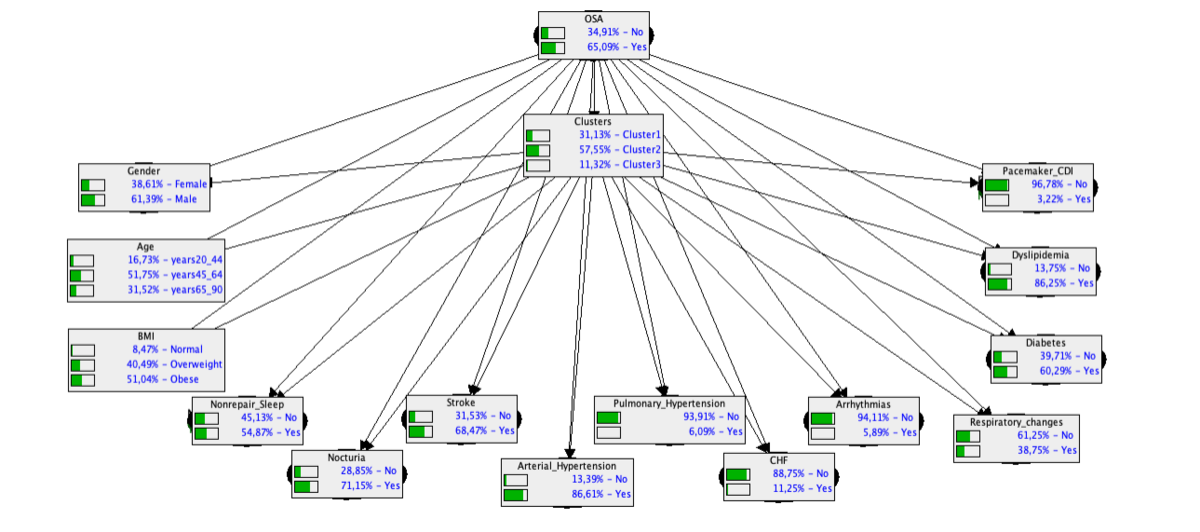
Naïve Bayesian network representation with additional node obtained from predefined obstructive sleep apnea phenotypes. The bars within each variable represent the prior marginal probabilities for the category of each variable. CDI: implantable cardioverter-defibrillator; CHF: congestive heart failure; OSA: obstructive sleep apnea.

**Figure 3:**
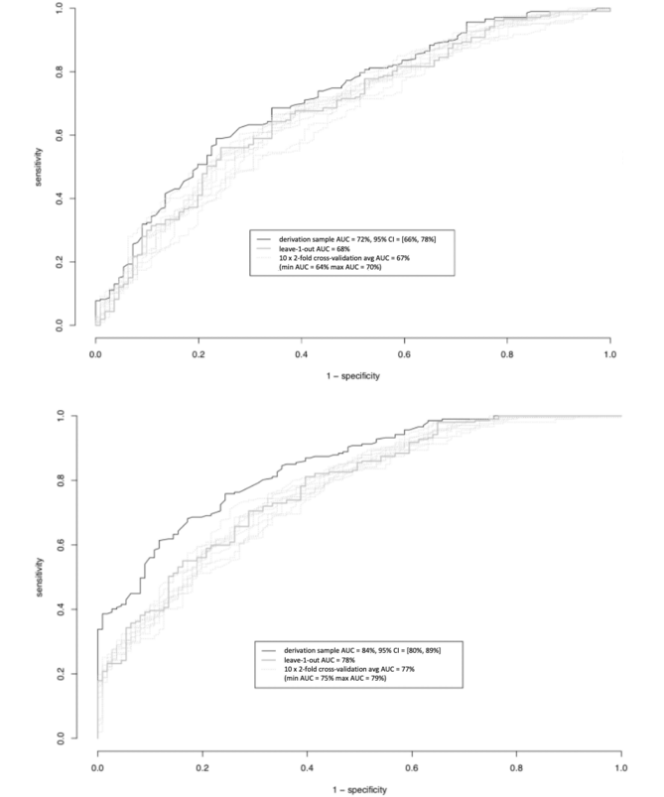
Receiver operating characteristic analyses and AUCs for models A (top) and B (bottom) as well for the internal validation procedures. AUC: area under the curve.

Initially, we created the simplest Bayesian classifier (naïve Bayes; Figure 1, Model A), which assumes independence among predictive variables and conditional independence, given the outcome. Subsequently, we extended the model (Figure 2, Model B), adding the defined phenotypes as a parent node of all predictors, thereby adjusting the model by capturing possible interactions among them, expressed by the corresponding phenotype associated with the tested individual. To evaluate the benefits of including OSA phenotypes in the clinical risk assessment tool, it was necessary to estimate the overall performance of each model. The ROC curves of each model (for both leave-one-out and cross-validation estimates) are presented in Figure 3, assessing the discriminative power of both models. As shown in Table 4, the derivation sample (area under the curve [AUC]) improved from 72% (95% CI 66-78) for model A to 84% (95% CI 80-89) for model B. The validity assessment confirmed the improvement achieved by the inclusion of OSA phenotypes, with leave-one-out estimates of 68% to 78%, respectively, from model A to model B and with 10 times twofold cross-validation averaging 67% and 77%, respectively. In addition, the diagnostic odds ratio, as a measure of the effectiveness of a diagnostic test, was 3.55 for model A and 2 times more for model B

**Table 4:** Validity assessment estimated from 10 times twofold cross-validation.

Variables	Obstructive sleep apnea	
	Model A	Model B
Cutoff point	30	22
Accuracy, % (95% CI)	69 (67-70)	74 (72-75)
Sensitivity, % (95% CI)	91 (89-94)	93 (91-95)
Specificity, % (95% CI)	26 (24-29)	38 (35-40)
Positive predictive value, % (95% CI)	70 (69-70)	73 (73-74)
Negative predictive value, % (95% CI)	64 (58-70)	75 (70-80)
Area under the curve, % (95% CI)	67 (67-70)	77 (76-78)
Positive likelihood ratio (95% CI)	1.32 (1.17-1.49)	1.63 (1.39-1.91)
Negative likelihood ratio (95% CI)	0.17 (0.09-0.34)	0.12 (0.06-0.22)
Positive odds posttest (95% CI)	2.45 (2.02-3.01)	3.02 (2.43-3.78)
Negative odds posttest (95% CI)	0.32 (0.19-0.56)	0.22 (0.12-0.38)
Posttest probability (95% CI)	71 (66-76)	75 (70-80)

Aiming at a 95% sensitivity target (screening strategies look for rule-out approaches), cutoff points were defined based on the derivation sample ROC curve, and the corresponding validity assessment results for cross-validation are displayed in Table 4, presenting an increase of specificity (26%-38%) for the desired level of sensitivity and presenting a posttest odds of 3 to 1 for the positive result and almost 1 to 5 for the negative result.

On the basis of the model with OSA phenotypes, OSA probabilities >22% were considered a positive result. The application of this cutoff resulted in a sensitivity value of 93% (95% CI 91-95) and 73% (95% CI 73-74) of positive predictive value, managing to provide a sensitive tool that prevents 1 out of 5 healthy individuals from unnecessarily undergoing PSG.

**Figure 4:**
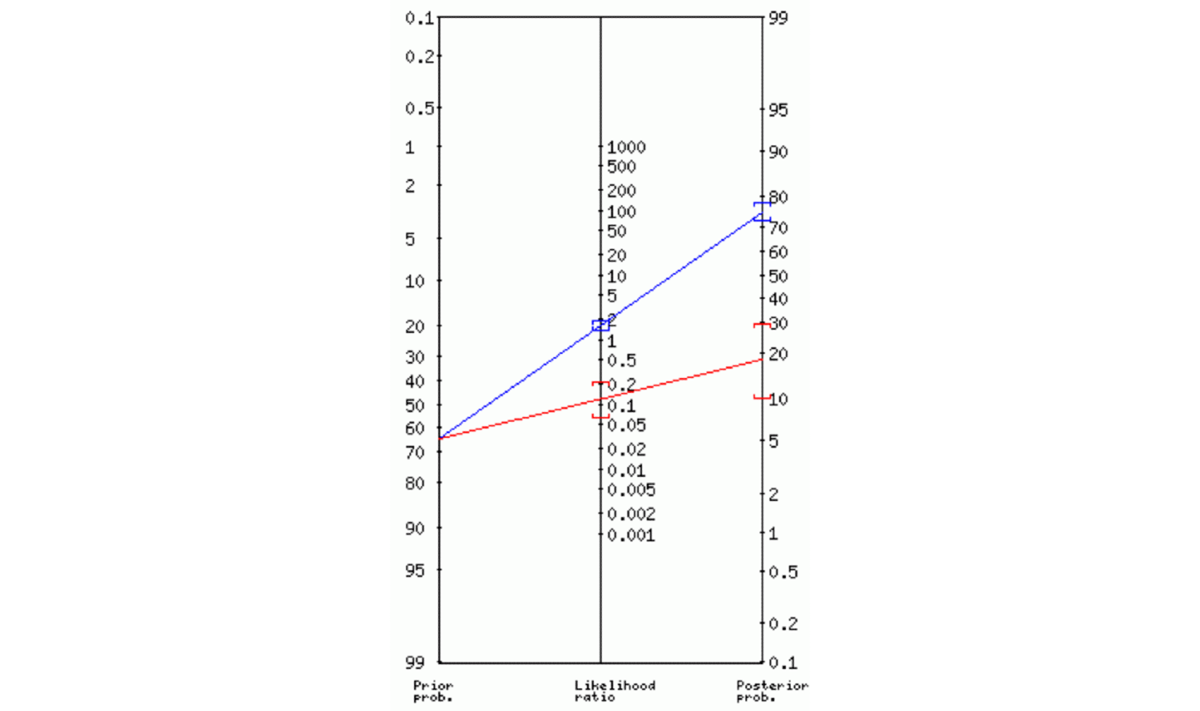
Fagan nomogram for model B. The blue and red lines represent the positive and negative posttest probability, respectively.

In our sample, the pretest probability was 65%, whereas the posttest probability increased to 75% using model B, with a posttest negative probability of 18%, as shown in Figure 4. These results highlight the value of using defined OSA phenotypes as predictors of OSA risk in referred individuals.

## Discussion

### Principal Findings

Understanding OSA patterns is important, particularly in the diagnosis of OSA. The AASM task force affirmed that the evaluation with clinical tools, such as clinical prediction algorithms, was less burdensome to the patient and physicians when compared with PSG. However, their low levels of accuracy and the likelihood of misdiagnosis must be weighted. Therefore, they proposed a clinical algorithm for the implementation of clinical practice guidelines for OSA. In the second step of this algorithm, the increased risk of moderate to severe OSA is measured by the presence of excessive daytime sleepiness and at least two of the following 3 criteria: habitual loud snoring, witnessed apnea or gasping or choking, or diagnosed hypertension. When we applied this moderate to severe risk in our data set (n=318), we found a sensitivity of 29%, a specificity of 68%, a positive predictive value of 50%, and a positive likelihood ratio of 0.875, showing possible benefits for a rule-out approach. However, considering the target of moderate to severe OSA identification, this approach revealed a very low level of sensitivity for a rule-in approach, which would be expected in this case.

To the best of our knowledge, this study is the first attempt to explore different clinical phenotypes of patients with OSA using categorical cluster analysis combined with Bayesian networks. We applied a hierarchical clustering procedure using Ward linkage on 14 significant predictive variables (out of the tested 47) that were grouped into 3 clusters: low, medium, and high severity phenotypes. These phenotypes were then used to expand a clinical prediction algorithm based on Bayesian networks, creating a simple but complete and updatable tool for OSA screening that can deal with missing information, based only on clinical and demographic variables, which have the main advantage of being easily available and quickly acquired by physicians.

Cluster analysis has been used in many medical conditions aiming to identify clinical phenotypes, as in the case of patients with asthma [16], where 5 clinical phenotypes illustrated the heterogeneity of the disease and relevant differences in treatment. Regarding OSA, clustering had been discussed as a possible helpful tool back in 1992, where the work of Tsuchiya et al [34] tried to apply cluster analysis in patients with OSA to overcome the stated overemphasis regarding obesity, which may have caused some physicians to overlook other potential factors that predispose this condition. They considered the apnea index (the standard at the time) and applied hierarchical clustering with average linkage, resulting in 2 clusters. The authors highlighted the controversy on the number of clusters, stating that “it should be essential to determine the number of clusters in a realistic way, and also to interpret the structures of clusters from a biologic standpoint.” Ye et al [35] collected demographic and survey data about sleep-related health issues (using numeric predictive variables) identifying 3 clusters: cluster 1 as *disturbed sleep group*, cluster 2 as *minimally symptomatic group*, and cluster 3 as *excessive daytime sleepiness group*. Although we have studied predictive variables related to daytime sleepiness, none were considered statistically significant; therefore, it is difficult to compare the results of the study by Ye et al [35] with the results of this study. Lacedonia et al [7] developed the work of Ye et al [35], enhancing the results using instrumental data, such as blood gas analysis and spirometry parameters (unavailable to us), to identify clinical presentations of patients with OSA. The authors used 2 approaches: a first one with hierarchical clustering revealing 3 clusters and the second one expanding it to 8 clusters with local optimization through principal component analysis.

Other studies are recently being developed, namely, the broad one in sleep apnea from the Sleep Apnea Network or European Sleep Apnea Database (ESADA) group. In 2016, Saaresranta et al [22] hypothesized that distinct OSA phenotypes should be present when discussing comorbidities and adherence to nasal continuous positive airway pressure (CPAP) therapy. This study has 3 main differences from ours: the ESADA database accepted PSG and cardiorespiratory polygraphy, whereas we only accepted PSG results; they accepted CPAP therapy and divided their patients into categories based only on subjective daytime sleepiness and nocturnal complaints. Regarding this last aspect, in our study, both subjective excessive daytime sleepiness and Epworth Sleepiness Scale were not considered in the cluster analysis. In 2020, a study by Bailly et al [21] applied latent class analysis to identify OSA phenotypes while reflecting geographical variations, resulting in 8 distinct clusters that were divided into 2 main categories: gender-based phenotypes (clusters 2 and 6 with only men and clusters 7 and 8 with only women) and men with various combinations (clusters 1, 3, 4, and 5), with which we can compare results. Cluster 3 of the study by Bailly et al [21] is described as obese comorbid patients, being the most similar to our low severity OSA cluster, presenting almost the same percentage of males (69% vs 73%) and higher levels of metabolic comorbidities.

Our results suggest 3 OSA phenotypes that can help in the screening, diagnosis, and later treatment of patients with OSA, capturing the full OSA spectrum of patients, focusing our attention on a detailed description of patients with OSA and not on a stereotypical one, where only a few *typical* symptoms such as snoring or daytime sleepiness are analyzed. To augment awareness of this prevalent disease, we even analyzed healthy patients to determine whether we could use the created phenotypes as identifiers of precursors of OSA.

### Strengths and Limitations

This study had a modest number of patients, mainly because of the short period for data collection, which was performed in a small district hospital. Nevertheless, we believe that the procedure and the results are relevant. We also acknowledge that our phenotypes are not fully in accordance with the clinical phenotyping experience, particularly those regarding upper airway morphology. We suppose that the inclusion of other relevant outcome data could create a more robust analysis of the determined phenotypes. The inclusion of more patients and even dissociating variables, such as craniofacial upper airway abnormalities, could benefit future research.

The major strengths of this study are the study of a clinical cohort representing patients with OSA with all levels of severity and the inclusion of a comprehensive number of risk and diagnostic factors that enhance our understanding of OSA diagnosis, with an overall cross-validated discriminative power of AUC of 77%, improving the specificity of a (designed) 95% sensitivity rule-out clinical prediction algorithm (3 to 1 odds for a positive result and 1 to 5 odds for a negative result). In addition, a diagnostic odds ratio higher than 1 was observed for models A and B, supporting the effectiveness of both models, with model B (inclusion of the disease phenotypes) doubling the diagnostic model performance. To assess the validity of our approach, we evaluated a logistic regression model in the derivation cohort, with and without predefined clusters, which highlighted the added discrimination value of using OSA phenotypes as a predictive variable (81% vs 83%). Moreover, we are aware that several clinical questionnaires (Berlin, STOP-BANG [snoring, tiredness, observed apnea, blood pressure, body mass index, age, neck circumference and gender], and NoSAS [neck, obesity, snoring, age, sex]) are helpful in identifying patients who are at risk of OSA. The Berlin questionnaire, when applied to the general population, reaches values of 37% for sensitivity and 84% for specificity, whereas when applied to primary care patients, the values are 86% and 77% [36], respectively. If we look at the STOP-BANG questionnaire, validation was performed in preoperative patients; the sensitivity and specificity values are 84% and 39%, respectively, for OSA diagnosis [37]. Finally, the NoSAS score was validated for the general population; the sensitivity values varied between 79% and 85%, the specificity varied between 69% and 77%, and AUC varied between 74% and 81% [38]. Comparing these results with our results, we can see that our sensitivity has the highest value, as we aim to establish a rule-out approach. On the other hand, our values for specificity and AUC were lower, only comparable with the value obtained for STOP-BANG.

### Conclusions

We can affirm that using OSA phenotypes as predictors allows the creation of sensitive tools, with the defined phenotypes being a reflection of the early expression and the natural history of OSA. Nevertheless, OSA and individual responses are not static and evolve with time, creating the need for further studies on evaluating the phenotyping fluctuations and determining their long-term diagnosis implications.

## Appendix

Multimedia Appendix 1 Descriptive analysis of patients with obstructive sleep apnea after missing data imputation (absolute and relative frequencies are presented, and <italic>P</italic> values are the result of chi-square tests unless otherwise specified). Footnote a: <italic>P</italic><.20; these values are italicized. Footnote b: Fisher exact test. Footnote c: the odds ratio was calculated for moderate and severe levels combined because of the absence of patients with normal abdominal circumference at the severe level.

## Abbreviations

AASM: American Academy of Sleep Medicine
AHI: apnea-hypopnea index
AUC: area under the curve
CPAP: continuous positive airway pressure
ESADA: European Sleep Apnea Database
NN: nearest neighbor
NoSAS: neck, obesity, snoring, age, sex
OSA: obstructive sleep apnea
PSG: polysomnography
ROC: receiver operating characteristic
STOP-BANG: snoring, tiredness, observed apnea, blood pressure, body mass index, age, neck circumference and gender
