# Supplementary figures and images for: Enhancing Obstructive Sleep Apnea Diagnosis With Screening Through Disease Phenotypes: Algorithm Development and Validation
Source: JMIR Med Inform. 2021 Jun 22;9(6):e25124. doi: 10.2196/25124 (PMC8277326; doi:10.2196/25124)

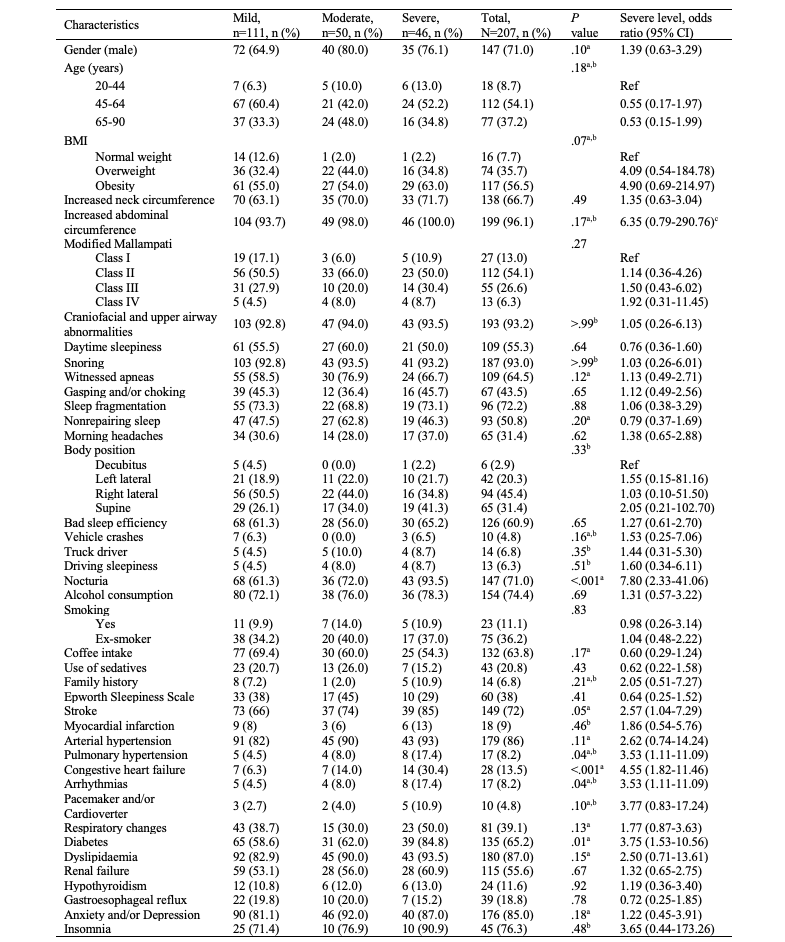

Supplement: Multimedia Appendix 1 [file medinform_v9i6e25124_app1.png]
